# Cell Migration Related to MDR—Another Impediment to Effective Chemotherapy?

**DOI:** 10.3390/molecules23020331

**Published:** 2018-02-05

**Authors:** Jakub Kryczka, Joanna Boncela

**Affiliations:** Institute of Medical Biology, Polish Academy of Sciences, 106 Lodowa Str, 93-232 Lodz, Poland; jkryczka@cbm.pan.pl

**Keywords:** ABC transporters, MRP, multidrug resistance in cancer, MDR reversal, metastasis, cell migration

## Abstract

Multidrug resistance, mediated by members of the ATP-binding cassette (ABC) proteins superfamily, has become one of the biggest obstacles in conquering tumour progression. If the chemotherapy outcome is considered successful, when the primary tumour volume is decreased or completely abolished, modulation of ABC proteins activity is one of the best methods to overcome drug resistance. However, if a positive outcome is represented by no metastasis or, at least, elongation of remission-free time, then the positive effect of ABC proteins inhibition should be compared with the several side effects it causes, which may inflict cancer progression and decrease overall patient health. Clinical trials conducted thus far have shown that the tested ABC modulators add limited or no benefits to cancer patients, as some of them are merely toxic and others induce unwanted drug–drug interactions. Moreover, the inhibition of certain ABC members has been recently indicated as potentially responsible for increased fibroblasts migration. A better understanding of the complex role of ABC proteins in relation to cancer progression may offer novel strategies in cancer therapy.

## 1. Introduction

Cancer cells have developed several mechanisms that allow them to survive and progress in human organisms and resist anticancer therapy. Those mechanisms consist of deregulation of growth regulatory pathways by acquiring growth factor independence, suppression of the immune system, low expression of antigens that activate T lymphocyte cells (mimicry), induction of anti-apoptotic signals that compensate for the pro-apoptotic effect of drugs and, finally, the active efflux of drugs from the cell cytoplasm [1]. Multidrug resistance, mediated by members of the ATP-binding cassette (ABC) proteins superfamily, has become one of the biggest obstacles in overcoming tumour progression. The overexpression of certain ABC proteins results in an apparent increase in carcinoma cell resistance to chemotherapeutics (e.g., paclitaxel, docetaxel, doxorubicin, cisplatin, topotecan, mitoxantrone (MTX), vincristine or vinblastine), leading to higher patient mortality. They protect cells through the active efflux of potentially harmful xenobiotics; thus, inhibition of their activity is considered a standard clinical approach. However, recent data suggest that inhibition of certain members of Multidrug Resistance Proteins (MRPs) inflicts severe side effects [2]. Furthermore, some additional findings showed that neoadjuvant chemotherapy, despite decreasing primary tumour volume, results in higher metastasis, leading to increased mortality [3]. Moreover, routinely used maximum dose chemotherapy, due to selection of ABC protein-overexpressing cells, cause not only tumour remission but also increase chemotherapy resistance in newly formed metastases [4]. Thus, MRPs may play a more complex role in cancer progression than previously considered.

## 2. ATP-Binding Cassette (ABC) Proteins—Structure and Function

The ABC proteins superfamily is one of the largest and most numerous groups of proteins, with more than 3000 members found among all species from archaebacteria to humans. All ABC proteins found in eukaryotic organisms are exporters; however, some ABC importers are present in prokaryotes. Among all 48 members of seven families of the human ABC proteins (ABCA–ABCG), members of three (ABCB, ABCC and ABCG) are involved in the active efflux of anticancer drugs from the cell cytoplasm [1,5,6,7]. They are also known by alternative names: Multi Drug Resistance protein 1 (MDR1) or P-glycoprotein (P-gp)—ABCB1; Multidrug Resistance Proteins (MRPs)—ABCC family; and Breast Cancer Resistance Protein/MitoXantrone Resistance-associated protein (BCRP or MXR)—ABCG2 [5,8]. All ABC proteins possess evolutionarily conserved structures of nucleotide binding domains (NBDs) and α-helical transmembrane domains (TMDs) [9]. The full structure is organized into four domains—2 × TMD (TMD1 and TMD2) and 2 × NBD (NBD1 and NBD2)—constituted by a single polypeptide. ABC proteins that show this composition are named full transporters (TMD1-NBD1-TMD2-NBD2 or NBD1-TMD1-NBD2-TMD2). Half transporters comprise only two domains, NBD-TMD or TMD-NBD, and form functional transporters either by homo- or hetero-dimerization. ABC proteins in eukaryotes are mainly represented by full and half transporters [9]. Nevertheless, two more groups can be distinguished: single structure transporters with only one domain (only NBD or TMD domain) and ABC2 structure composed of NBD-NBD [9,10]. NBD domains consist of 200–220 aa, and their most conserved regions are called the Walker A and B motifs, the signature region (LSGGQ motif, linker peptide or C-loop) and the D, H and Q loops. The binding of ATP in the presence of Mg^2+^ is mediated by the Walker A and B motifs of one NBD and the C- and D-loop of the second [11]. Hydrolysis of ATP on the NBD domains drives conformational changes in the TMD domains, resulting in alternating access from inside and outside of the cell for unidirectional transport across the cell membrane [10]. Additional data suggest that ATP binding rather than hydrolysis is sufficient to trigger NBD dimerization and substrate transport [12]. ABC transporters that are “pumps” that actively transport substrates against a gradient have their NBDs function as ATPases (Mg^2+^ ATP + H2O → Mg^2+^ ADP + Pi); however, some that form channels in which anions flow passively require energy input. There is a question regarding the stoichiometry in the relationship between the number of ATP molecules hydrolysed and the number of substrate molecules transported, as the number of ions flowing through the channel bears no fixed stoichiometric relationship to ATP hydrolysis. ABCC7, also known as CFTR (cystic fibrosis transmembrane conductance regulator), is a model example. It forms an ion channel and functions as an adenylate kinase (Mg^2+^ ATP + AMP ↔ Mg^2+^ ADP + ADP), where ATP actually stabilizes the structure of an active channel rather than propelling transport [13]. Furthermore, several ABCC family transporters (ABCC1, -2, -3, -4 and -8) use glutathione (GSH) to increase or enable the transport of several substrates. They transport GSH conjugates with higher affinity [14,15,16,17] or use them as stimulators. The stimulatory effect of GSH can be explained by three different models: (a) co-transport of GSH and substrate (S)—in which neither one can be transported alone; (b) heterotrophic cooperativity—when GSH and S are transported with a higher KM than each alone; and (c) ”membrane effect”—in which GSH alters the membrane environment of the transporter, increasing or enabling its affinity to S [18]. Nevertheless, all ABC transporters use ATP either to actively transport substrates or to stabilize the channel conformation.

## 3. ABC Transporters from Discovery to Clinical Testing

The role of ABC proteins in xenobiotic transport has been known for some time. In 1974, V. Ling and L.H. Thompson showed that colchicine-resistant lines selected and isolated from Chinese hamster ovary (CHO) cells displayed cross-resistance to other drugs, such as actinomycin D, vinblastine and colcemid. Furthermore, they showed that this phenomenon was related to the presence and activity of ABCB1 [19]. Further research showed that CHO origin cell lines resistant to daunorubicin also present cross-resistance to a variety of other drugs, demonstrating that this mechanism, related to P-glycoprotein expression, could be the basis of many drug-resistance phenotypes observed in vivo [20]. The next ground-breaking step came in 1992, when S.P. Cole published her studies showing that another ABC protein (later known as ABCC1) was responsible for chemotherapeutic agent resistance in the doxorubicin-selected lung cancer cell line H69AR, which does not express P-gp [21]. The term “multidrug resistance (-associated) protein” (MRP) was first used in 1994 to relate overexpression of ABCC1/MRP1 with increased resistance to several anticancer therapeutics (doxorubicin, daunorubicin, epirubicin, vincristine and etoposide) and since then has been used to refer to almost all ABCC family drug resistance related transporters [14,22,23]. Until 1998, only two subfamilies of ABC transporters (ABCB and ABCC) were thought to be involved in drug resistance phenomena. However, studies conducted on MCF-7 mitoxantrone-resistant sublines of breast cancer cells, in the absence of the overexpression of known multidrug resistance transporters, revealed a new ATP-dependent pump of anthracycline anticancer drugs named breast cancer resistance protein (BCRP)—now known as ABCG2 [24,25,26]. Since then, members of the ABCB, ABCC and ABCG families have been reported to cover with their substrate, specifying a vast range of anticancer and antiviral chemotherapeutics, and named “multidrug ABC transporters”. Their current nomenclature is presented in Table 1.

### 3.1. The Largest Multidrug Resistance Protein Family (MRP)

MRPs are the largest subfamily of drug efflux-related transporters and share a highly similar transport mechanism. As mentioned in Table 1, they all belong to the ABCC family. They have been shown to mediate the efflux of several anticancer and antiviral drugs, e.g., thiopurines, methotrexate (MTX), camptothecins, 9-(2-Phosphonylmethoxyethyl)adenine (PMEA) and azidothymidine (AZT) [27], and can be found in most tumours and normal tissues [28]. Furthermore, 5-Fluorouracil (5-FU) is considered as a possible ABCC4 substrate. It was demonstrated that the rs3742106 polymorphism in the 3′-UTR that enhances miR-3190-5p-mediated inhibition of ABCC4 expression is significantly related to the increased efficacy of 5-FU/capecitabine-based chemotherapy in colorectal cancer [29]. As revived by Zhang et al., numerous reports of drug resistance in patients emphasized the upregulation of MRP proteins in vast numbers of cancers: MRP1, MRP4 and MRP8 in breast cancer; MRP1-4 in lung cancer; MRP1–3 in non-small cell lung cancer; MRP1 in pancreatic cancer; MRP1 and MRP4 in kidney cancer; and MRP1, MRP2, MRP4, MRP6 and MRP8 in colorectal cancer [28]. Moreover, in a group of patients with metastatic colorectal cancer, chemotherapy outcome was correlated with elevated transcript levels of the MRP6 and MRP8 genes [30]. Furthermore, functional consequences of Mrp gene knockout in mouse models (Abcc−/−) demonstrated increased sensitivity to anticancer drugs, e.g., etoposide phosphate, doxorubicin, topotecan, 2-chloro-2′-deoxyadenosine (2CdA), and MTX. All of the above-mentioned data demonstrated the necessity for MRP inhibition to overcome drug resistance in cancer treatment [31,32,33,34,35]. However, Hlavata et al. states that, despite many published studies conducted on both animals and cell models, there are no convincing data that could clinically serve to predict ABC protein levels and chemotherapy outcomes in patients, leaving to further discussion of the not fully understood importance of ABC proteins as markers of cancer metastasis [30]. The gene expression of ABCC9 is significantly increased in metastases compared to primary carcinomas in epithelial ovarian cancer, suggesting it as a possible useful marker [36]. Surprisingly, increased levels of ABC transporters from non-multidrug-related subfamily A (ABCA1, ABCA6, ABCA8, and ABCA9) in primary tumours were statistically significantly associated with reduced survival in ovarian cancer patients [37]. Furthermore, despite several in vitro studies of ABCG2 involvement in drug efflux and drug resistance, recent in vivo data showed no connection between ABCG2 expression and drug resistance [38] as well as no relationship between a patient´s progression-free survival or overall survival and level of ABCG2 expression in ovarian carcinoma patients [37].

### 3.2. Reversal of Multidrug Resistance

The inhibition of multidrug ABC transporters activity is considered to be one of the major anticancer strategies. Several pharmacological agents have been tested positive to inhibit drug resistance. Commonly, they are named multidrug resistance inhibitors (due to high protein homology, they usually target several ABC transporters from different subfamilies); however, this term is misleading as they range from small molecules, which interact with the 3D structure of the transporter to block substrate recognition by competitive binding, to antibodies and tyrosine kinase inhibitors, which target the PI3K-Akt pathway to downregulate ABC expression, up to miRNA, siRNA and shRNA, which regulate expression of ABC genes [28,39,40,41,42]. Thus, a more adequate name would be multidrug resistance modulators. Since ABCB1 was discovered in the mid-1970s, the first attempts of clinical usage of ABC protein modulators in the 1980s were focused on this protein. However, the first generation of multidrug resistance modulators that target the ABCB1 substrate binding site (verapamil and cyclosporine A) were found to be quite toxic and immunosuppressive at doses required to inhibit its in vivo activity, thus failing clinical trials [43]. Furthermore, in the mid-1980s, the first antibodies against ABCB1 were generated—MRK16, which modulates vincristine and actinomycin D transport in human myelogenous leukaemia K-562 cells resistant to adriamycin (K-562/ADM) [44]. Since then, MRK16 has been widely employed to study P-gp structure and function in drug discovery and development research. The second generation inhibitors—valspodar PSC 833 (ABCB1), dexaverapamil (ABCB1), and biricodar VC 710 (ABCB1 ABCC1 and ABCG2) showed hardly moderate effects during phase II and III clinical trials, with no significantly enhanced antitumor activity or patient survival rate [45,46,47,48]. The third generation of modulators, tariquidar (XR9576), ontogen (OC144-093), zosuquidar (LY335979), elacradir (GF120918), and dofequidar, were specially designed and synthetized to improve second generation selectivity and decrease toxicity, as they were reported to work at nanomolar concentrations. They are currently under clinical trials, with proven in vitro effectiveness against ABCB1- and ABCG2-mediated drug resistance [42,43,49,50,51,52]. ABCB1 expression has been shown in many cancer types; however, ABCB1 modulation therapy has not been effective against tumours that possess ABCC family-mediated drug resistance, as shown for the doxorubicin-selected lung cancer cell line H69AR and HL60/AR [53]. Thus, the first attempts to increase cancer cell chemosensitisation by modulation of MRP activity were performed in vitro with verapamil and NIK250 [54]; however, as previously reviewed, verapamil failed clinical trials. In comparison, the ABCC family inhibitor MK571, which was first discovered in 1995, presents low intrinsic toxicity, with potent inhibitory properties [55], becoming the “gold standard” for MRP modulators. Furthermore, in 1996, probenecid, a known inhibitor of multispecific organic anion transport, was first reported to inhibit ABCC1-mediated basolateral transport, but not ABCB1-mediated apical transport of daunorubicin in ABCB1-transfected polarized pig kidney epithelial cell line LLC-PK1 [56]. Probenecid was also effective in ABCC1-positive HL60/AR and H69/AR cells but not in ABCB1-overexpressing HL60/Tax and P388/ADR cells [57], presenting promising results for future clinical ABCC/MRP modulation [56]. Moreover, the multi-kinase inhibitor sorafenib (BAY 43-9006) was reported to decrease the mRNA levels of ABCB1 and ABCC1-3 in hepatocellular carcinoma (HCC), suggesting the involvement of the RAF/MEK/ERK pathway in the regulation of multidrug resistant cancer cells [58]. Further analysis revealed that the MEK inhibitors U0126 and AZD6244 modulated the MAPK pathway by increasing the p-MEK levels and decreasing p-ERK levels, downregulating the expression of ABCC1 and ABCC3 in HCC [59]. Nevertheless, the next generation of MRP modulators will use antisense-, ribozyme-, RNA interference- and/or CRISPR/Cas9-based techniques to impair ABC protein formation [60]. A “multitarget multiribozyme” (MTMR) containing three trans-acting hammerhead ribozymes that, after autocatalytic self-activation, cleave the transcripts of the ABC transporter genes ABCB1, ABCC2, and ABCG2 tested positive in several cancer cell models [61]. Moreover, several siRNAs were designed for gene-silencing RNA interference (RNAi) targeting major multidrug resistance proteins (ABCB1, ABCC2–3, and ABCG2) [42]. Next, as the siRNAs showed biological activity for stable inhibition of ABCC2 and ABCG2, corresponding short hairpin RNA (shRNA) vectors were designed (shRNA shows the highest efficiency in gene silencing). Treatment of human ovarian carcinoma A2780RCIS (ABCC2) and human gastric carcinoma EPG85-257RNOV (ABCG2) cell lines with this construct inhibited expression of the targeted encoding mRNA and protein itself [62,63]. Moreover, several miRNAs were recently selected by microarray techniques. miR-297 was reported to directly inhibit ABCC2 expression at the post-transcriptional level through its 3′-UTR in the multidrug resistant variants of the CRC cell line HTC-8 and HTC116. A similar effect was reported for ABCC1, as the expression levels of the mRNA and protein were reduced in the miR-326 miRIDIAN mimic-transfected VP-16-resistant breast cancer cell line MCF-7/VP [28]. Furthermore, recent studies have shown that RNAi-based methods of overcoming multidrug resistance are effective not only in vitro but also in vivo in mouse models. Nude mouse xenografts were injected with a novel vector pEGFP-H1/mdr1 containing Mdr1-shRNA targeting at position 2943–2963 of Mdr1, which reduced the expression of Mdr1 mRNA and Mdr1 protein [64]. The most commonly used and tested modulators of multidrug resistance associated ABC proteins are shown in Table 2.

### 3.3. Physiological Aspects of ABC Protein Modulation

Multidrug ABC transporters, transport not only xenobiotics but also a variety of endogenous amphiphilic organic anions and cations conjugated to reduced glutathione (GSH), glucoronate, and sulphate ore phosphate [84]. Inhibition or modulation of MRP activity increases sensitivity towards anticancer drugs; however, it can lead to severe collateral damage affecting normal functions of cells, e.g., inflammatory response, bile acid secretion or testosterone production [2].

Multidrug ABC transporters participate in the inflammatory response, ABCB1 transports platelet activating factor [85], and MRP4 transports prostaglandins [86], whereas all MRPs, except for MRP5, transport leukotriene C4 (LTC4) [87,88]. Naturally, in humans, pro-inflammatory interleukin 1beta (IL-1β) represses the mRNA expression of several anion channels, including MRP2, MRP3 and MRP4 [89]. Furthermore, LPS-mediated inflammation decreases Abcb1, Abcc4 and Abcg2 proteins expression and upregulates Abcc1 and Abcc5 in mouse BV-2 microglial cell models [90]. Microglia play a prominent role in brain inflammation and neurodegenerative diseases, and disruption of ABC transporters function in activated microglia may alter cell-cell communication and cause chemical sequestration in the brain [90]. Inhibition of ABC activity leads to impaired secretion of pro-inflammatory substances; the broad range ABCC inhibitors, probenecid and MK571 decreased the acute inflammatory response induced by zymosan (a chemical agent used to induce experimentally sterile inflammation) in mice [91]. Acute inflammation is most often associated with neutrophil-rich cellular infiltration and is generally resolved in a period of days [92]. Furthermore, cellular accumulation of prostaglandin (PGE2) inflicted by ABCC4 inhibition decreases cell migration by lowering COX-2 expression and β-catenin nuclear translocation, impacting wound healing time and inflammation, as presented in human skin explant dendritic cells (DCs). MRP4 is needed for optimal DC migration toward the lymph node-homing chemokines CCL19 and CCL21, and its inhibition reduced the amount of migrated skin DCs by 60–70% [85,93]. Moreover, decreased cell recruitment may prolong healing time and increase the chance of fibrosis-related disorders [94].

Several ABC proteins share partially identical substrate species, allowing for transport compensation, e.g., both ABCC2 and ABCC3 mediate the efflux of bilirubin diglucuronide, decreasing the possibility of tissue self-poisoning. Inactivation of ABCC2, which is mainly located in the apical membrane of hepatocytes, substantially increases ABCC3 protein expression, as observed in an Abcc2-deficient rat model [95]. Furthermore, ABCC3 basal expression is very low in comparison to ABCC2, but as the expression of these two genes is regulated inversely, ABCC3 level increases in negative feedback [96]. However, ABCC3 is mainly located in the basolateral membrane, thus severely impairing bile acid secretion. Bilirubin diglucuronide instead of bile is transported into blood, causing Dubin-Johnson syndrome [97,98]. Furthermore, ABCC3 and ABCC4 serve as back-up mechanisms for bile salt export pumps (BSEPs) on the basolateral membrane. Impaired BSEP-mediated hepatic bile acid export during ABC protein inhibition may contribute to the development of cholestatic drug-induced liver injury (DILI) [99]. Broad range MRP inhibitors, e.g., probenecid, verapamil or MK571, can overcome substrate compensation by modulating ABC transporter-mediated secretion activity, leading to intrinsic accumulation of toxic compounds in the liver or kidneys [97,98,99].

MRP4, a thiopurine nucleotide exporter, is expressed in the plasma membrane of Leydig cells, which are the primary source of testosterone production in males [100]. Impairment of ABCC4 function decreases testosterone production and its serum concentration in a cAMP-dependent manner. Heterozygous MRP4+/− mice present 50% serum testosterone concentration in comparison to homozygous MRP4+/+, whereas MRP4−/− mice present only approximately 20% serum testosterone concentration. Decreased MRP4 activity attenuates cAMP-response element binding protein (CREB) phosphorylation, leading to alterations of genes that contain CREP binding sites in the promoter region and are related to testosterone biosynthesis—StAR and 3-β-HSD [2]. Furthermore, data obtained from adult survivors of childhood acute lymphoblastic leukaemia (ALL), which were treated with 6-mercaptopurine (6 MP), suggest that therapeutics that disrupt MRP4 function can alter androgen production [100]. The combination of 6 MP and MTX is a main component of ALL treatment; however, many patients require a dose reduction of 6-MP due to its severe toxicity. Patients with a high level of MRP4 protein present less intolerability to 6 MP treatment [101], and MRP4-mediated efflux of cAMP decreases apoptosome formation in Leydig cells, leading to their protection and thus unimpaired testosterone production [100].

Mrp6-deficient mice show ectopic mineralization of connective tissues (skin, arterial blood vessels and retina) affecting both elastic structures and collagen fibres, similar to pseudoxanthoma elasticum (PXE) observed in patients [88,102]. According to recent studies, PXE is caused by impaired ABCC6-mediated ATP efflux (which is extracellularly converted into AMP and PPi). Deficiency of the potent anti-calcifying molecule PPi circulating in blood causes excessive mineralization [103,104]. This mechanism confirms that modulation of MRP activity in one tissue may impact distinct organs, as ABCC6/Abcc6 expression is almost entirely missing in both normal and PXE affected tissue in humans, mice and rats; however, its downregulation in the liver causes PXE [104].

One of the most important functions of ABC proteins as efflux pumps is the creation of the blood–brain barrier (BBB) and the blood–cerebrospinal fluid barrier (BCSFB), which maintain brain homeostasis by eliminating metabolic waste products and preventing the uptake of both endogenous and exogenous potentially harmful substances [105,106,107]. The BBB is composed of a monolayer of brain microvessel endothelial cells joined by tight junctions to create an impermeable barrier surrounded by pericytes, astrocytes, and neurons. They express several ABC transporters, with the most important being ABCB1, ABCC1, ABCC4 and ABCG2 [105,108]. Inactivation of ABC protein-mediated xenobiotic efflux increases the concentration of chemotherapeutics in the brain, as shown in the model of MRP4-deficient mice (Abcc−/−) that presented enhanced accumulation of topotecan [34], MTX, raltitrexed and cyclophosphamide [108]. Global administration of MRP modulators during anticancer therapy expose delicate neuronal tissue (that is not a primary target) to cytotoxic properties of the drugs leading to severe damage, e.g., depressed hippocampal cell proliferation and increased cognitive impairment implicated by MTX [109].

## 4. Benefits and Downsides of ABC Modulation in Cancer

Cancer cells originate from normal cells that acquire the first cancer-promoting mutation(s); thus, anticancer therapies are extremely difficult and cause numerous side effects as they affect both the cancer and normal cells [110]. Anticancer monotherapy, which uses single chemotherapeutics, causes drug resistance in patients after several cycles of treatment. Thus, the combination of multi-chemotherapeutic agents with synergistic effects increases the chances of success and is the most commonly used strategy [111].

Side effects caused in normal cells affected by global multidrug resistance inhibition were balanced by anti-cancer properties in preclinical studies [43]. Elacridar (GF120918), which is an ABCG2 and ABCB1 inhibitor, increased the oral bioavailability of topotecan from 40% to approximately 100% in randomized trials on different cancer patients (non-small cell lung cancer, small cell lung cancer, ovarian adenocarcinoma, appendix carcinoma, pancreas carcinoma, squamous cell carcinoma, bladder carcinoma, and gastric carcinoma) [112,113]. Reversan, the small molecule ABCB1 and ABCC1 inhibitor, which presents no toxic effects by itself, increased the efficacy of vincristine and etoposide chemotherapy in murine models of neuroblastoma [80]. Furthermore, it was shown that depletion or inhibition of ABCC4 can inhibit cell growth of neuroblastoma cells [114] and reduce proliferation of pancreatic cancer [115], not only increasing sensitivity to chemotherapeutics but also presenting cytostatic abilities. Moreover, the small molecule tyrosine kinase (TK) inhibitor imatinib (Glivec, Gleevec, STI571) used for the treatment of BCR-ABL-positive chronic myelogenous leukaemia or acute lymphoblastic leukaemia, despite being reported to be a substrate for ABCB1 and ABCG2 (with several reports stating that it is also transported by ABCC1), seems to increase the intracellular concentration of other ABC protein substrates in cancer cells: for example, combined with vincristine, enhanced vincristine sensitivity of MDR K562 cells (which overexpress ABCB1) in a human nude mice xenograft model. This effect is probably obtained due to high affinity to ABC transporters, which locally increases other substrate concentrations [116]. In addition, nilotinib (AMN107, Tasigna), another TK inhibitor used in the treatment of BCR-ABL-positive chronic myelogenous leukaemia, also transported by ABCB1, ABCC10 and ABCG2, significantly enhanced the cytotoxicity of colchicine, vinblastine and paclitaxel in KB-C2 and KB-V1 cells, showing that the combination of different anticancer agents is likely to have an additional synergistic beneficial effect [117]. Surprisingly, ABC proteins overexpression during cancer progression may become a positive factor, as small molecule thiosemicarbazone NSC73306 was reported to kill cells with ABCB1-mediated multidrug resistance, as shown for several cancer cell lines, thus indirectly eliminating resistance to MDR1 substrates [118]. Furthermore, a similar effect was observed in both in vitro (epidermal carcinoma-derived cell line KB-3-1, the promyelocytic leukaemia cell line HL60, the non-small cell lung cancer cell lines A549, VL-6 and VL-8, the small cell lung cancer cell line GLC-4, the glioblastoma U373, the hepatocellular carcinoma cell line Hep3B, and the breast cancer cell lines MCF7 and MDA-MB-231) and in vivo (human colon carcinoma xenograft) models overexpressing ABCB1, ABCC1 or ABCG2 proteins for lanthanum compound KP722. KP722 induced a higher amount of apoptosis and cell cycle G0/G1 arrest in ABC-overexpressing cells compared to normal cells, highlighting a new possible means of overcoming drug resistance [119,120]. However, some authors doubt that inhibition of ABC transporters can effectively overcome drug resistance in vivo [121]. Thus far, clinical trials have shown that tested multidrug resistance modulators add limited or no benefits to cancer patients, as some of them are merely toxic and others induce unwanted drug–drug interactions [122]. Recently, professor M. H. Oktay’s group observed that breast cancer spread to other parts of the body when three specific cells are in direct contact: (1) endothelial cells; (2) a Tie2-Hi perivascular macrophages; and (3) tumour cells, creating a site called a tumour microenvironment of metastasis (TMEM). They demonstrated that chemotherapy increases the density and activity of TMEM sites, thus promoting distant metastasis. Moreover, in the residual breast cancers of patients treated with neoadjuvant therapy paclitaxel after doxorubicin and cyclophosphamide, the TMEM score was elevated, suggesting that chemotherapy, despite decreasing tumour size, increases the risk of metastatic dissemination [3]. Furthermore, it is known that after a certain period of remission-free time following a high dose of traditionally used chemotherapy, newly formed metastases present higher drug resistance [4]. Moreover, tamoxifen, the primary metabolite 4-hydroxy-*N*-desmethyl tamoxifen, is an ABCB1 substrate [123] widely used in breast cancer therapy (oestrogen receptor-positive subtype); however, 50% of patients develop resistance after 5 years of treatment, increasing risk of metastasis related to an invasive tumour phenotype acquired by epithelial to mesenchymal transition (EMT) [124]. In cancer patients, EMT-derived circulating tumour cells (CTCs) (CK/E-cadherin negative, vimentin/N-cadherin positive) are chemoresistant [125]. Furthermore, despite breast cancer patients being treated with poly-chemotherapy, including MDR and MRP inhibitors, metastatic progression of the disease occurred, with a staggering 82.3% of patients being completely resistant to neoadjuvant therapy [121]. These data triggered an alarm regarding whether currently used chemotherapy and multidrug resistance inhibition contributes to increased metastasis by selecting drug-resistant cells, generating a higher number of secondary tumours after the period of remission.

## 5. Non-Canonical Activity of ABC Transporters in Cell Migration

Cell migration is a very composed process, regulated by many often, ostensibly not migratory related signalling pathways, that allow cells to dynamically adjust to microenvironment changes inflicted by a variety of factors (e.g., growth factors, cytokines, mediators of inflammation, physical/mechanical irritation, etc.) [126]. Thus, it has become a cutting-edge field in cancer research. Cell migration comprises five consecutive steps: (1) extension of the leading edge with formation of pseudopod; (2) adhesion to matrix contacts; (3) translocation of the cell body via contraction of the cytoplasm; (4) release from the rear contact sites; and (5) recycling of membrane receptors from the rear to the front of the cell [127]. There are two distinct cell migration models that are applied to motile cells: an ameboid and a mesenchymal type [128]. The ameboid type of migration is characterized by a spherical shape (blebby ameboid migration type) or by a slightly elongated cell shape with actin rich filopodia. The ameboid migration of cells is characterized by a three times higher velocity (0.6–0.8 µm/min) than the mesenchymal (0.2 µm/min) type. Moreover, it is strongly dependent on Rho/ROCK (Rho-associated kinase) activity, phosphorylation of myosin II, and it is independent of extracellular matrix (ECM) degradation and cell adhesion [126]. On the other hand, mesenchymal type migration exhibits elongated cell morphology with long protrusions. It is strongly dependent on Rac kinase activity, proteolytic degradation of ECM components and adhesion via integrins [126]. Both small GTPases Rho and Rac belong to the Ras family; however, they exert opposite effects. Rho increases cell contractility, whereas Rac intensifies actin polymerization. Regulatory proteins that favour the amoeboid mode either activate RhoA/ROCK signalling or inhibit Rac activation. Proteins that act in the antiamoeboid mode activate Rac signalling [129]. However, intermediate model between ameboid and mesenchymal has recently been proposed, suggesting that cells may shift between the types of migration according to many factors, such as changes in microenvironment plasticity, loss of close cell-cell connections (tight junctions), availability of the vascular endothelial growth factor (VEGF) isoforms as observed for fibrosarcoma [130] or chemokines and growth factors released by inflammatory cells as reported for microvascular endothelial cells [126,131]. Migration type shift enables effective migration in changing environments (changes in ECM substrates, breaching anatomical boundaries, etc.) that are encountered during metastasis.

**Figure 1:**
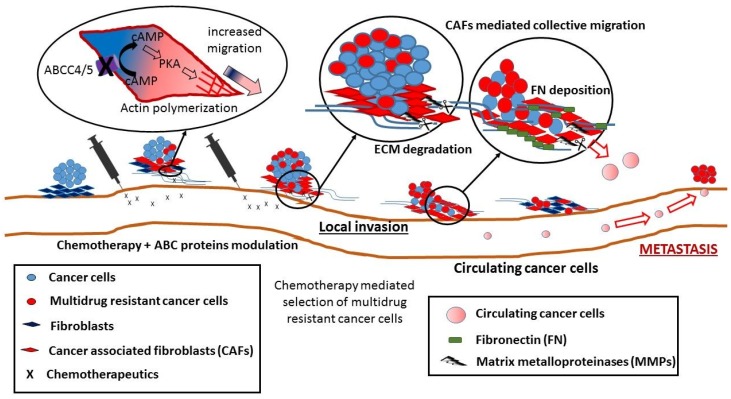
Putative effect of ABCC4/5 modulation on cancer metastasis. Inhibition of ABCC4/5 mediated cyclic nucleotide transport increases cAMP intracellular concentration, leading to enhanced actin polymerization, via cAMP-dependent protein kinase (PKA), and resulting in a gain in the high migratory abilities by cancer associated fibroblasts (CAFs) [136] and cancer cells [149,150]. Chemotherapy mediates tumour regression but increases the number of multidrug resistant cancer cells. CAFs forms an “advance guard” on the leading edge of invading cancer cells and process extracellular matrix (ECM) to prepare a metastatic niche. Red arrows indicate cell migration, black arrows—enlarged part of the scheme.

MRP4, MRP5 and MRP8 influence numerous nucleotide-dependent pathways by controlling the cyclic nucleotide concentration and may actually regulate cell migration. Inhibition of their activity either by MRP modulators or by substrate competition decreases the efflux of cAMP (KM = 45 µM) and cGMP (KM = 10 µM), increasing their cytosolic concentration [132,133]. Several drugs used in anticancer or antiviral therapy, e.g., methotrexate, leucovorin, tenofovir or adefovir, exhibit ten times higher affinity to ABCC4 or ABCC5 than cyclic nucleotides, decreasing availability of the free transporter, thus acting as MRP modulators [84,132,133]. The effect of cyclic nucleotides on cell migration was thought to be well established, with both nucleotides possessing opposite effects, i.e., cAMP inhibits and cGMP stimulates cell migration [134]. The formation of lamellipodia, during cell direct migration, at the leading edge of mouse embryonic fibroblast cells and mouse breast tumour cells was shown to be inhibited by cAMP, which acts downstream of the small GTPase, Rac, whereas cGMP plays the opposite role in the modulation of lamellipodium formation [135]. However, their actual role in the regulation of cell migration appears to be much more complicated and controversial. Recent studies showed that the increased concentration of cAMP at the leading edge is responsible, via cAMP-dependent protein kinase (PKA) actin polymerization, for increased cell direct migration of ABCC4 −/− fibroblasts [136]. On the other hand, cGMP-dependent protein kinase (PKG) downregulation, observed in hypoxia conditions of pulmonary vascular smooth muscle cell, increases their migration and integrin mediated adhesion. Moreover, in normoxia conditions, higher migration was observed upon PKG inhibition [137]. It is suggested that cyclic nucleotides do not regulate migration by their higher or lower concentration, but rather by changes in the balance between them. Inhibition of ABCC4 activity by MK571 resulted in the increased migration of fibroblasts [133], similar results were obtained by siRNA silencing of ABCC4 in human retinal microvascular endothelial cells (HRECs) [138]. The effect of ABCC4 modulation on fibroblast migration is completely abolished by disruption of the actin cytoskeleton or inhibition of PKA. Moreover, MRP4−/− fibroblasts present more polarized activity of PKA, focused mainly on the leading edge of cells [136,139]. Interestingly, F-actin has been identified as a downstream target of MRP4 and a major mediator of its impact on cell migration with cAMP/cGMP playing the role of signalling molecules [139]. F-actin plays a crucial role in the formation of invadosomes—key structures for cells that are able to cross anatomical boundaries like metastatic cancer [126]. Cancer associated fibroblasts (CAFs) that originate from normal stromal tissue surrounding the tumour [4] are the major sources of chemokines and growth factors in the tumour microenvironment [140] both secreted and released during extracellular matrix (ECM) degradation [94]. Their ability to secrete TGF-Beta and HGF promote EMT-derived cancer progression [141,142]. However, both fibroblasts and CAFs may shift their migration model [126] and by an active modulation, degradation and rearrangement of ECM in the tumour surrounding enhance tumour angiogenesis and promote directional cancer cell migration [143,144,145,146]. CAFs were reported to lead invasion of various cancer types: squamous cell carcinoma, melanoma, colon and scirrhous gastric carcinoma. Their secreted matrix-degrading enzymes (such as matrix metalloproteinases—MMPs) cleave ECM and base membrane on the invading front [141,142,147,148] forming tracks in the extracellular matrix and allowing for collective migration of cancer cells. CAFs invasion on the leading edge of the metastatic cancer depends on Rho-mediated regulation of myosin light chain assembly and invadosome activity [126,149]. Furthermore, CAFs co-injected with noninvasive PROb cells (rat colon carcinoma derived cell line) into syngeneic BD-IX rats facilitated their invasive abilities. Moreover, investigation of cells from a freshly dissociated PROb tumour showed not only PROb cells but also CAFs mainly located at the invasive edge of the tumour that were able to cross a Matrigel-coated filter [150]. Moreover, the cancer microenvironment is often named the “wound that does not heal”, in which more motile fibroblasts/CAFs are enforced to secrete certain ECM components (collagen type I and II, fibronectin), leading to formation of CAFs aggregates and matrix stiffening [142,145]. Most cells, including cancer cells, tend to migrate towards stiffer matrix [151]. CAFs secreting fibronectin-rich ECM, as presented in human prostatic and pancreatic carcinoma samples, have an anisotropic fibre orientation, which guides the cancer cells to migrate directionally through α5β1 integrin, thus leading to local invasion and metastasis [143]. MRP inhibition that increases migration abilities of fibroblasts and CAFs shows direct repercussions on metastasis (Figure 1); however, it is unknown whether the cAMP/cGMP-dependent mechanism of increased migration is actually present in cancer cells. The squamous cell carcinoma cell lines KYSE140 and KYSE180 present a lower migration rate and colony formation in a ABCC4 copy number related manner; however, this mechanism is related to cellular accumulation of PGE2 rather than cAMP/cGMP [152]. Nevertheless, cyclic nucleotide transporters were connected with higher metastasis, as patients treated with oxaliplatin- and 5-FU-based regimen for metastatic colorectal cancer CRC (*n* = 40) had a significantly shorter metastasis free period in the case where their CTCs expressed MRP5 [30].

## 6. Conclusions

Overcoming multidrug resistance that increases during cancer progression by modulating ABC protein activity has become one of the most important issues during chemotherapy. Observed secondary side effects must be balanced by the necessity of anticancer therapy success, according to the rule: exitus acta probat (the outcome justifies the deed). However, in vitro, the increase in the accumulation of chemotherapeutic agents upon ABC protein inhibition in cell lines expressing a single ABC transporter failed to take into consideration the physiological functions of the transporters in a whole organism and the possible co-expression of many transporters within tumours and neighbouring tissues [43]. Furthermore, a recent study suggested that inhibition of MRP activity can increase cell motility by cAMP/cGMP accumulation and the PKA/PKG signalling pathway [136]. Although this effect was reported mainly on non-cancer cells (fibroblasts), we hypothesize that this mechanism may be generalized to other cells. It is yet unknown whether this mechanism is present in cancer cells; however, increased migration of CAFs directly promotes cancer progression and metastasis [149,150]. Furthermore, higher MRP expression is related to the more advanced stages of cancer progression and correlates with both local and distant metastasis [30,36]. Thus, we hypothesize that the high-dose chemotherapy connected with ABC protein inhibition may act as a selective factor, killing tumours but possibly creating small, yet highly motile, aggressive and resistant populations of circulating cancer cells that contribute to increased metastasis, generating a higher number of secondary tumours after remission (Figure 1). Therefore, a new strategy for overcoming drug resistance needs to be applied, reducing all negative effects and focusing on targeting drug delivery.

## Figures and Tables

**Table 1:** Multidrug resistance associated ABC protein nomenclature.

ABC Gene	Alternative Names
*ABCB1*	MDR1 P-gp (P-glycoprotein 1)
*ABCC1*	MRP1
*ABCC2*	MRP2 cMOAT
*ABCC3*	MRP3 cMOAT-2
*ABCC4*	MRP4 MOAT-B
*ABCC5*	MRP5 MOAT-C
*ABCC6*	MRP6
*ABCC10*	MRP7
*ABCC11*	MRP8
*ABCC12*	MRP9
*ABCG2*	BCRP1 MXR1

**Table 2:** ABC proteins modulators (inhibitors).

ABC Protein Activity Modulator	Target	Effect
Biricodar	ABCB1 ABCC1 ABCG2	direct interaction [65]
Celecoxib	ABCC1	COX-2 inhibitor [66]
Curcumin	ABCB1 ABCC1 ABCG2	interacts directly with drug binding site of the transporter [67]
Cyclosporine A	ABCB1 ABCC1 ABCC2 ABCC10 ABCG2	interacts directly with drug binding site of the transporter [65,68]
Dexaverapamil	ABCB1	interacts directly with drug binding site of the transporter [69]
Dipiridamole	ABCB1 ABCC1 ABCC4	phosphodiesterase inhibitor [70]
Dofequidar	ABCB1 ABCC1 ABCG2	direct interaction [71]
Elacradir	ABCB1 ABCG2	direct interaction [65]
Indomethacin	ABCC1 ABCC2	COX and glutathione-S-transferase inhibitor, direct ABC protein inhibition [72]
Losartan	ABCB1 ABCC4	direct interaction [73,74]
MK571	ABCC family ABCG2	LTC4 receptor antagonist [55,75]
MRK-16	ABCB1	Antibody [44,76]
Ontogen	ABCB1	direct interaction [49]
Piperine	ABCB1 ABCC1 ABCG2	reduces ATPase activity of ABCB1 at high concentration and stimulates it at low concentration, decreases the expression level of *ABCB1*, *ABCC1* and *ABCG2* genes [77]
Probenecid	ABCC family	an organic anion transport inhibitor [56,78]
Quercetin	ABCC family	Interact with ATP binding site (NBD) [79]
Reversan	ABCB1 ABCC1	small molecule inhibitor [80]
Sildenafil	ABCB1 ABCC4 ABCG2	PDE5 inhiitor [70,81]
Sorafenib	ABCB1 ABCC1-3	multi-kinase inhibitor, downregulates *ABC* mRNA [58]
Tariquidar	ABCB1 ABCC1 ABCC10 ABCG2	interacts the transporter but not with drug binding site [51,52,82]
Valspodar	ABCB1 ABCC2	interacts directly with drug binding site of the transporter [75,83]
Verapamil	ABCB1 ABCC1	interacts directly with drug binding site of the transporter [65]
Zosuquidar	ABCB1	direct interaction [50]

Table modified from Ween et al. 2015 [43] and Yu et al. 2015 [39]
